# Supplementary material for: MYG1 drives glycolysis and colorectal cancer development through nuclear-mitochondrial collaboration
Source: Nat Commun. 2024 Jun 11;15:4969. doi: 10.1038/s41467-024-49221-0 (PMC11167044; doi:10.1038/s41467-024-49221-0)
Supplement: Supplementary file 1 — Supplementary Information [file 41467_2024_49221_MOESM1_ESM.pdf]

Supplementary Information for

MYG1 drives glycolysis and colorectal cancer development through nuclear-mitochondrial collaboration

Jianxiong Chen[1,2,#], Shiyu Duan[1,2,#], Yulu Wang[1,2], Yuping Ling[1,2], Xiaotao Hou[1,2], Sijing Zhang[1,2],Xunhua Liu[1,2], Xiaoli Long[1,2], Jiawen Lan[1,2], Miao Zhou[2], Huimeng Xu[1,2,] Haoxuan Zheng[3,*], Jun Zhou[1,2, *]

This file includes:

Supplementary Figures 1-8

Supplementary Tables 1-2

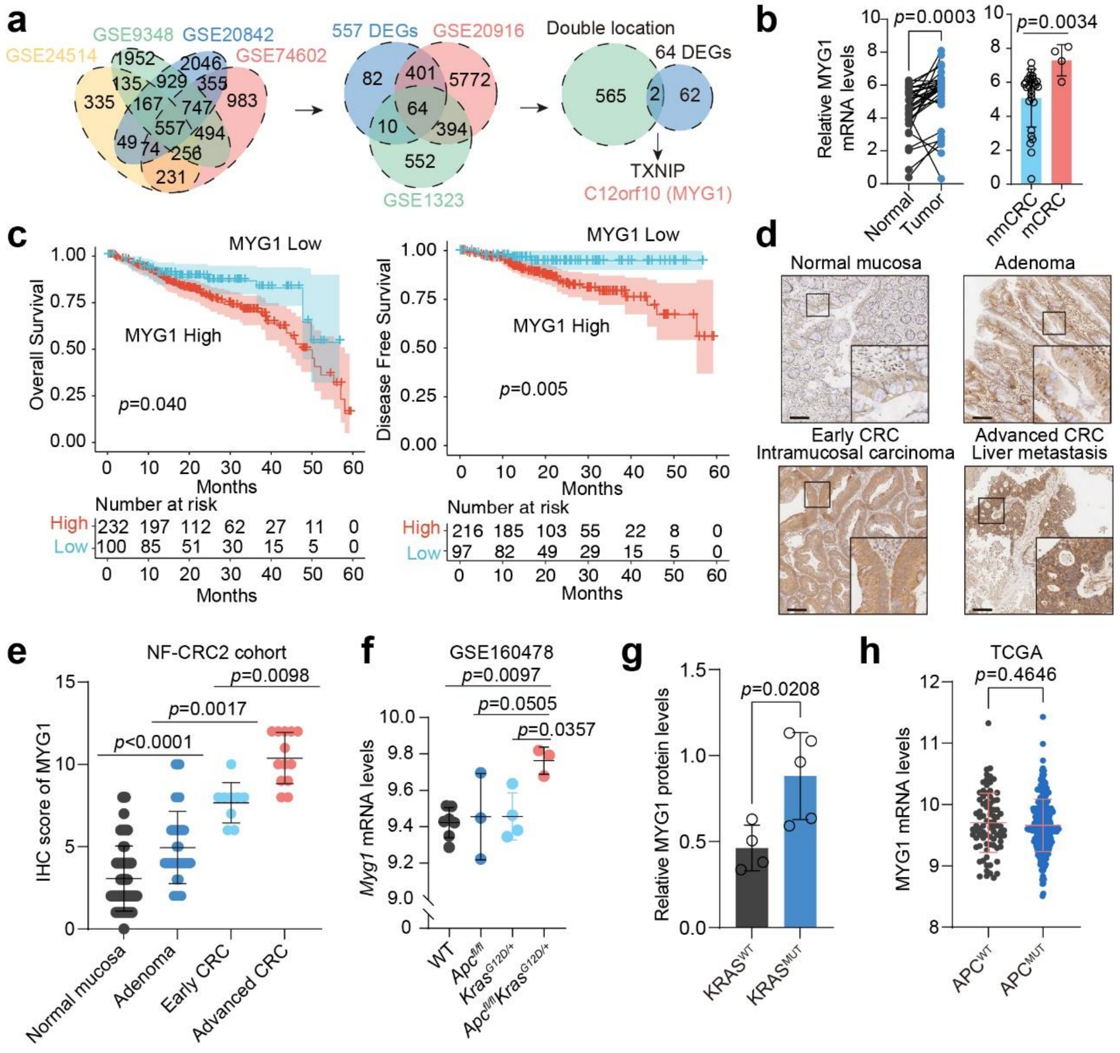

Supplementary Fig. 1. MYG1 is an oncogenic gene associated with CRC progression and poor clinical outcomes. Related to Fig.1. a Schematic of screening gene with dual nuclear and mitochondrial location related to CRC progression. **b** The relative mRNA expression in tumor and paired adjacent normal tissues (n = 31 pairs) (left), nmCRC (n = 27) and mCRC (n = 4) (right). nmCRC, CRC patients with no distant metastasis; mCRC, CRC patients with distant metastasis. **c** Kaplan-Meier analysis of five-year overall survival (left) and disease-free survival (right) of TCGA COADREAD patients (n =332). **d-e** Representative IHC staining images of MYG1 in normal mucosa (n = 64), adenoma (n = 38), intramucosal carcinoma (n = 9), and distant metasstasis (n = 13) in NF-CRC2 cohort (**d**) and quantification (**e**). Scale bar, 200 μm (main macrophages). **f** The mRNA levels of *Myg1* in mice with different *Apc*/*Kras* status in GSE160478 (WT, n = 7; $Apc^{fl/fl}$, n = 3; $Kras^{G12D/+}$, n = 4; $Apc^{fl/fl}Kras^{G12D/+}$, n = 3). **g** The quantificaiton of relative MYG1 expression in cell lines with different KRAS status (Related to **Fig. 1f**). **h** Transcription levels (estimated in $\log_2$ (x+1) transformed RSEM normalized count) of

MYG1 in TCGA COADREAD tumor samples with APC wild type (WT, $n = 82$) and mutation (MUT, $n = 275$). Unpaired two-sided Student's t-test (**g** and **h**), One-way ANOVA, Tukey's multiple comparisons test (**e** and **f**). Log-rank test (**c**). Mann-Whitney test (**b**). p value was provided in the figure. Data represents the mean ± SD. Representative results were shown (**d**). Source data are provided as a Source Data file.

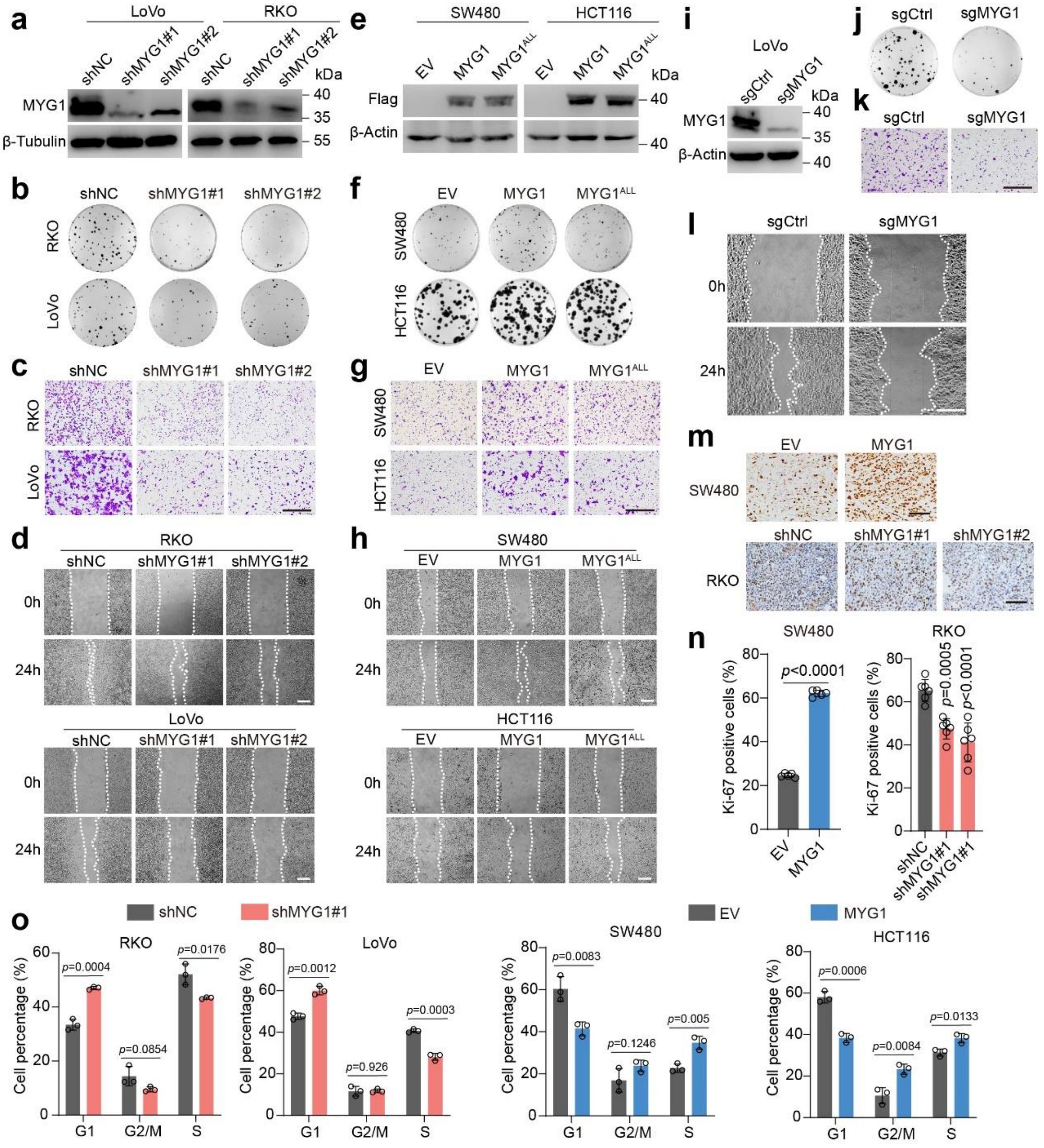

Supplementary Fig. 2. MYG1 accelerates CRC proliferation and metastasis in vitro and in vivo. Related to Fig. 2. a-i MYG1 was knocked down in RKO and LoVo cells (**a**), overexpressed in SW480 and HCT116 cells (**e**), and knocked out in LoVo cells (**i**) as confirmed by western blot, respectively. Representative images of colony formation assay (**b, f,** and **j**), transwell invasion assay (**c, g,** and **k,** Scale

bar, 200 μm) and wound healing assay (**d, h,** and **l**, Scale bar, 200 μm). **m-n** Representative IHC staining images of Ki-67 in tumors from subcutaneous xenograft model in Fig. 2**m** (*n* = 5 in SW480 and *n* = 6 in RKO) (**m**) and quantification (**n**). Scale bar, 200 μm. **o** CRC cells as indicated were examined for cell cycle distribution by flow cytometry. *n* = 3 independent experiments (**a-l** and **o**). Unpaired two-sided Student's *t*-test (**n**, and **o**). One-way ANOVA, Tukey's multiple comparisons test (**n**). *p* value was provided in the figure. Data represents the mean ± SD (**n**, and **o**). Source data are provided as a Source Data file.

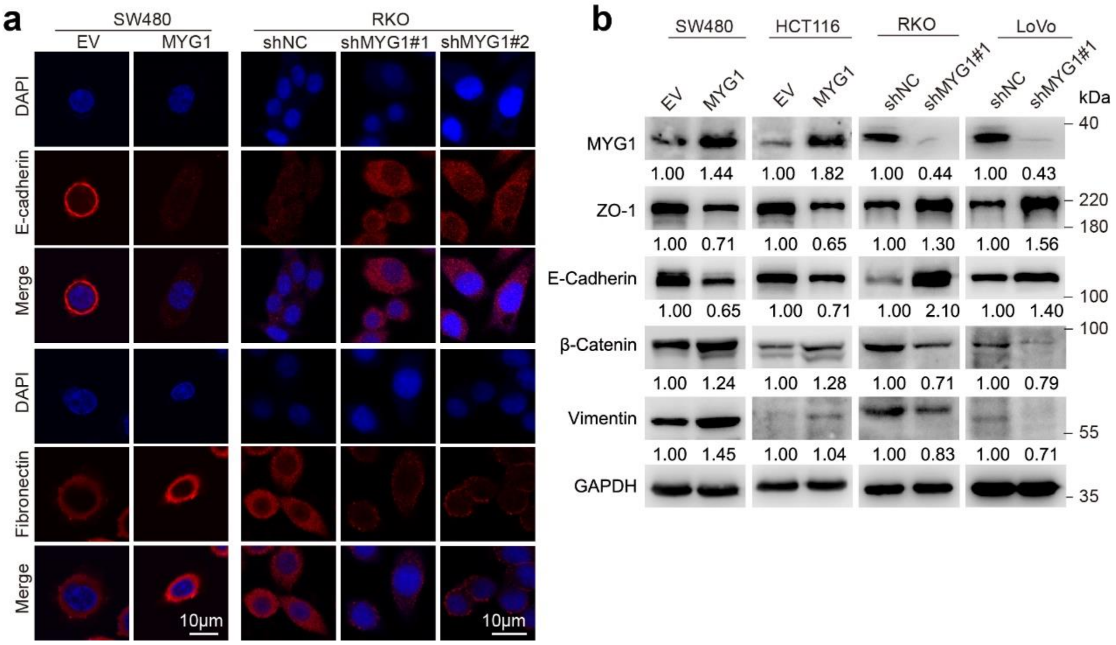

Supplementary Fig. 3. MYG1 accelerates EMT of CRC cells. Related to Fig. 2. a CRC cells as indicated were examined for E-Cadherin and Fibronectin by western blot. Scale bar, 10 μm. Representative images from *n* = 3 independent experiments. **b** CRC cells as indicated were examined for epithelial (E-Cadherin and ZO-1) and mesenchymal (β-Catenin and Vimentin) markers by western blot. *n* = 3 independent experiments. Source data are provided as a Source Data file.

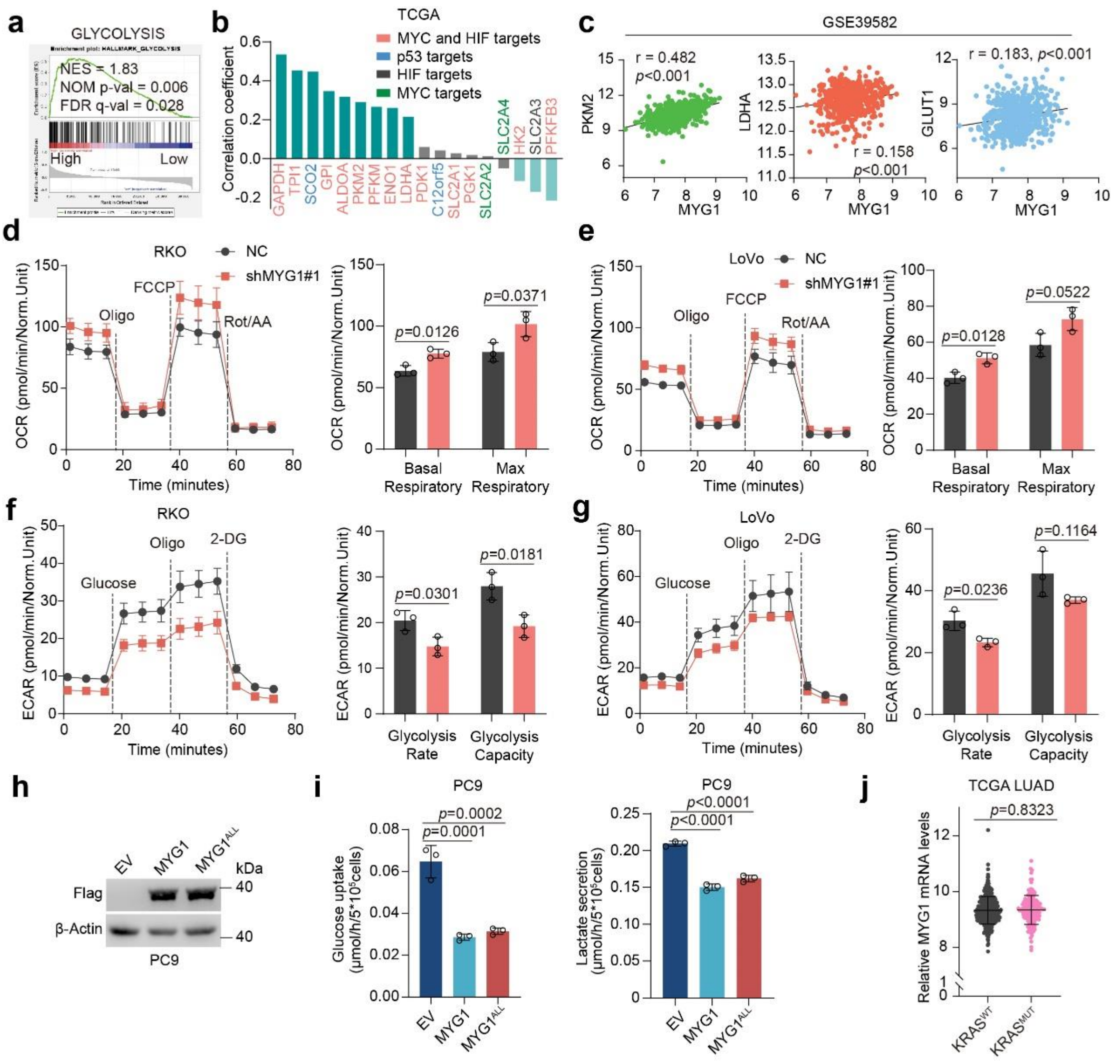

Supplementary Fig. 4. MYG1 promotes aerobic glycolysis of CRC cells in vitro. Related to Fig. 3. **a** Glycolysis pathway is enriched in MYG1 highly expressed CRC patients from TCGA COADREAD cohort. **b** Correlation between the expression of MYG1 and key glycolysis-related genes in TCGA COADREAD cohort based on transcriptome. **c** Correlation between the expression of MYG1 and key glycolysis-related genes (PKM2, LDHA, and GLUT1) in CRC patients from GSE39582 datasets (n = 585). **d-g** OCR (**d** and **e**) and ECAR (**f** and **g**) were examined in RKO (**d** and **f**) and LoVo cells (**e** and **g**) with MYG1 stably knocked down (left) and quantified (right). Norm.Unit represents the normalized OCR and ECAR. **h-i** Lung adenocarcinoma cell line PC9 was transfected with Flag-tagged MYG1 and MYG1^{ALL}, and confirmed by western blot (**h**) and then glucose uptake and lactate secretion were detected (**i**). **j** MYG1 mRNA levels in patients with different KRAS status in the TCGA LUAD cohort (WT, n = 361; MUT, n = 148). n = 3 independent experiments (**d-i**). Unpaired two-sided Student's t-test (**d-g,** and **j**). One-way ANOVA, Dunnett's multiple comparisons test (**i**). Pearson correlation (**b** and **c**). p

value was provided in the figure. Error bars, mean ± SD. Source data are provided as a Source Data file.

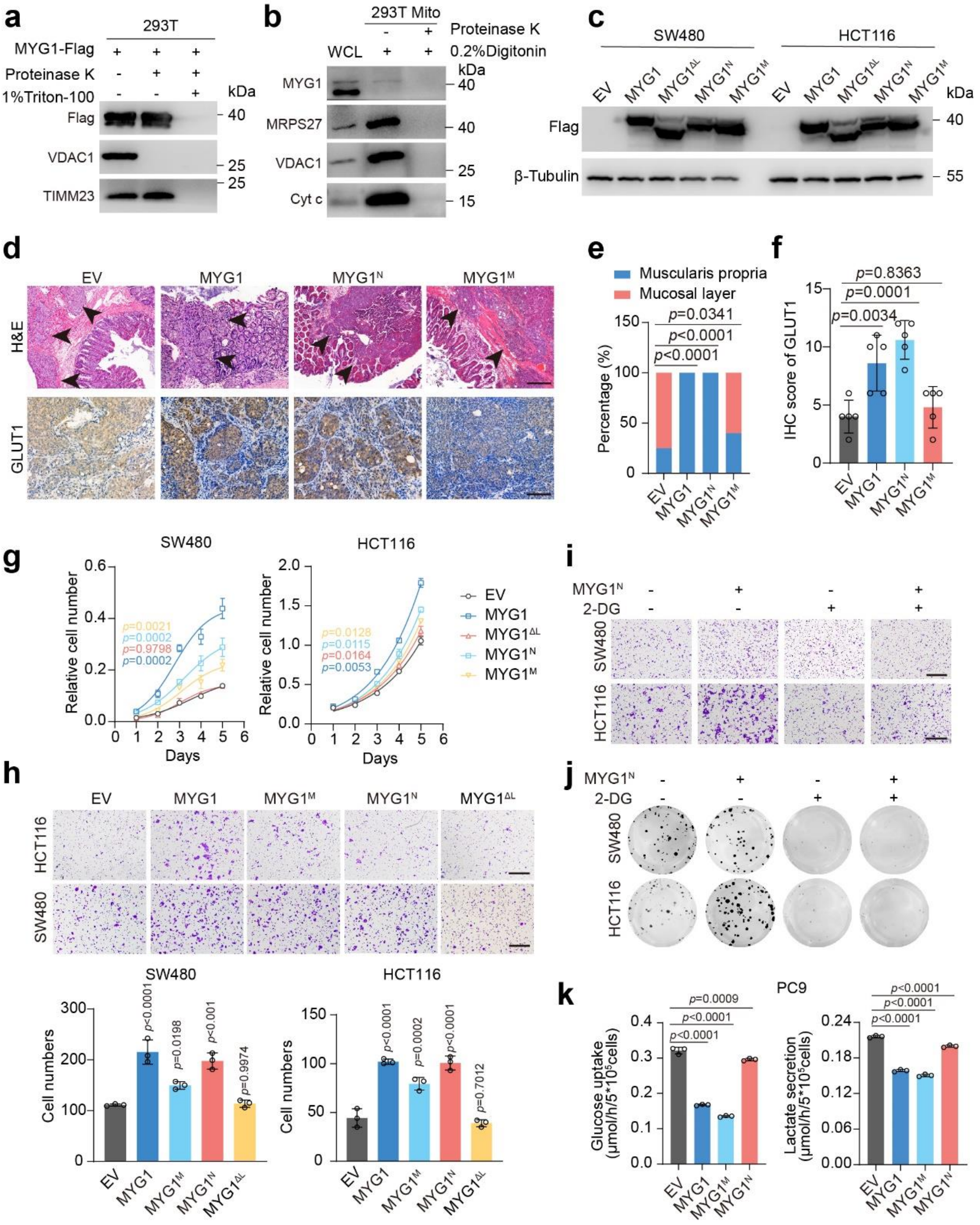

Supplementary Fig. 5. The nuclear localization of MYG1 plays a dominant role in promoting CRC progression through glycolysis. Related to Fig. 4. a Mitochondrial fraction from 293T cells transfected with Flag-tagged MYG1 was analyzed by protease K shaving assay. VDAC1 was sensitive to protease K treatment, whereas TIMM23 was resistant. Proteins were digested by protease K in the presence of 1% Triton X-100. **b** Purified mitochondria from 293T cells were collected and analyzed by protease K

shaving assay. Proteins were digested by protease K in the presence of 0.2% digitonin. **c** Exogenous MYG1 protein in SW480 and HCT116 cells expressing Flag-tagged MYG1 variants were detected by western blot. **d-f** Representative images of H&E (top, scale bar, 400 μm) and GLUT1 IHC staining (bottom, scale bar, 200 μm) in tumors from orthotopic CRC models in **Fig. 4g-k (**left**)**. The invasion layer of tumor was analyzed (**e**) and IHC staining of GLUT1 was quantified (**f**). **g-h** Proliferation and invasion ability were examined in HCT116 and SW480 cells expressing different MYG1 variants by proliferation assay (**g,** $n = 3$ technical replicates, representative data from $n = 3$ independent experiments) and transwell invasion assay (**h,** scale bar, 200 μm), respectively. p value represents comparison with the EV group (**h**). **i-j** Representative images of transwell invasion assay (**i,** scale bar, 200 μm) and colony formation assay (**i**) in SW480 and HCT116 cells as indicated. **k** The levels of glucose uptake and lactate secretion in PC9 cells expressing different MYG1 variants. Fisher's exact test (**e**). $n = 3$ independent experiments (**a-c** and **h-k**). One-way ANOVA, Dunnett's multiple comparisons test (**f,** **h**, and **k**). Two-way ANOVA, Tukey's multiple comparisons test (**g**). p value was provided in the figure. Error bars, mean ± SD. Source data are provided as a Source Data file.

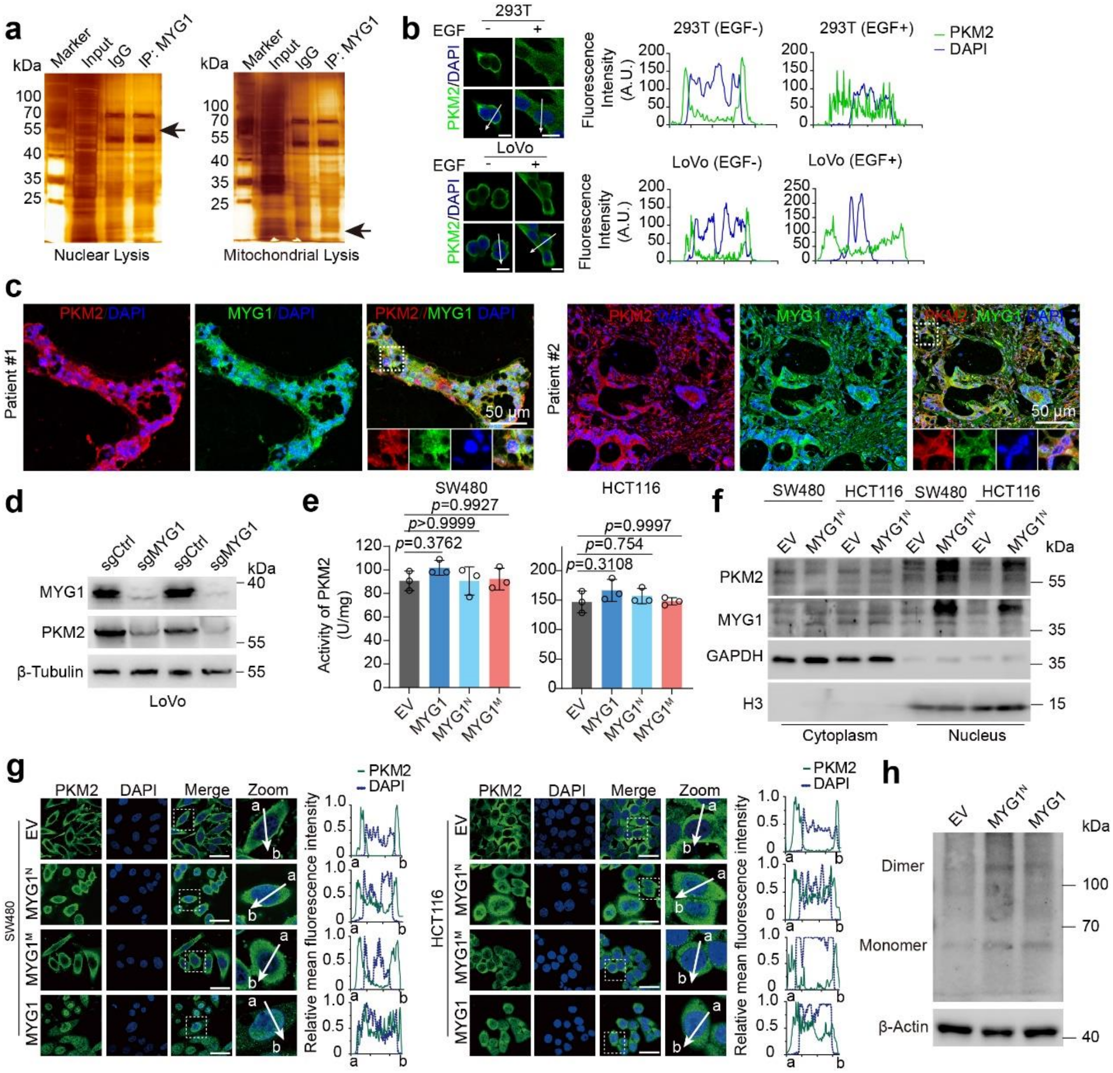

Supplementary Fig. 6. Nuclear MYG1 regulates PKM2 protein expression. Related to Fig. 5. a Nucleus and mitochondrion of SW480 cell overexpressing MYG1 were fractioned and subjected to immunoprecipitation using anti-MYG1 antibody and followed by SDS-PAGE. The sliver staining gels were shown and arrows represented proteins that interacted with MYG1 and cut for IP/MS. **b** Representative IF images of PKM2 in 293T and LoVo cells treated with EGF (100 ng/mL) for 10 h (left, scale bar, 10 μm.) and fluorescence density analysis (right). PKM2 protein (green) and nucleus (blue). Representative images from $n = 2$ independent experiments. **c** Representative IF images of MYG1(red), PKM2 (green), DAPI (blue) in CRC specimens with KRAS mutation from $n = 3$ patients. Scale bar, 50 μm. **d** PKM2 expression was detected by western blot in control and MYG1 KO LoVo cells. The blots showed two repeated experiments. **e** SW480 and HCT116 cells expressing MYG1 variants were detected for pyruvate kinase activity. **f** SW480 and HCT116 cells overexpressing MYG1^{N} were fractioned into cytoplasm and nucleus followed by western blot. **g** Representative IF images of PKM2

(green) and nucleus (blue) in SW480 and HCT116 isogenic cells (EV, MYG1, MYG1^{N}, MYG1^{M}). The areas marked by squares were magnified. Relative mean fluorescence intensity of PKM2 and DAPI on white arrow was shown on right. Scale bar, 20 μm. Representative images from n = 2 independent experiments. **h** SW480 isogenic cells (EV, MYG1, and MYG1^{N}) were examined for PKM2 dimer and monomer by western blot. The samples derive from the same experiments but different gels for PKM2, and another for β-Actin were processed in parrallel. n = 3 independent experiments (**d-f**, and **h**). One-way ANOVA, Dunnett's multiple comparisons test (**e**). Error bars, mean ± SD. Source data are provided as a Source Data file.

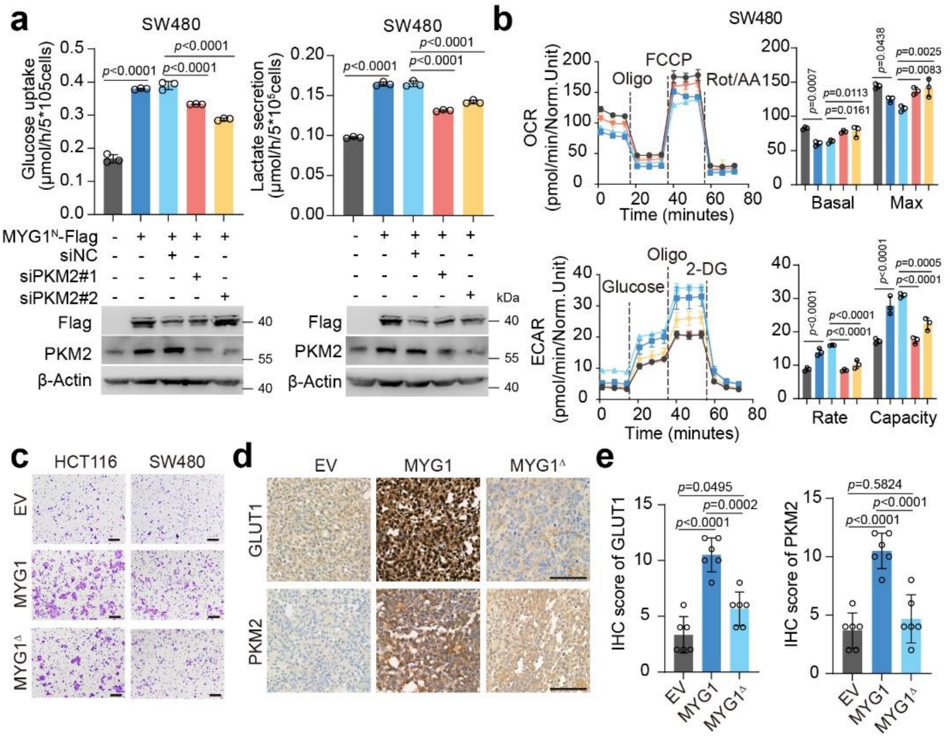

Supplementary Fig. 7. Nuclear MYG1 accelerates glycolysis through PKM2. Related to Fig. 6. a-b HTC116 cells overexpressing MYG1^{N} were transfected with siRNA of control (siNC) or PKM2 (siPKM2#1, siPKM2#2). Glucose uptake and lactate secretion (**a**), OCR and ECAR (**b**) were detected in cells as indicated. Norm.Unit represents the normalized OCR and ECAR. **c** Representative images of transwell invasion assay in HCT116 and SW480 cells expressing MYG1 or MYG1$^{\Delta}$, Scale bar, 100 μm. **d-e** Representative IHC staining images of GLUT1 and PKM2 in tumors from subcutaneous xenograft model in Fig. 6f (n = 6 in each group) (**d**, Scale bar, 100 μm) and quantification (**e**). n = 3 independent experiments (**a-c**). One-way ANOVA, Tukey's multiple comparisons test (**a, b,** and **e**). p value was provided in the figure. Error bars, mean ± SD. Source data are provided as a Source Data file.

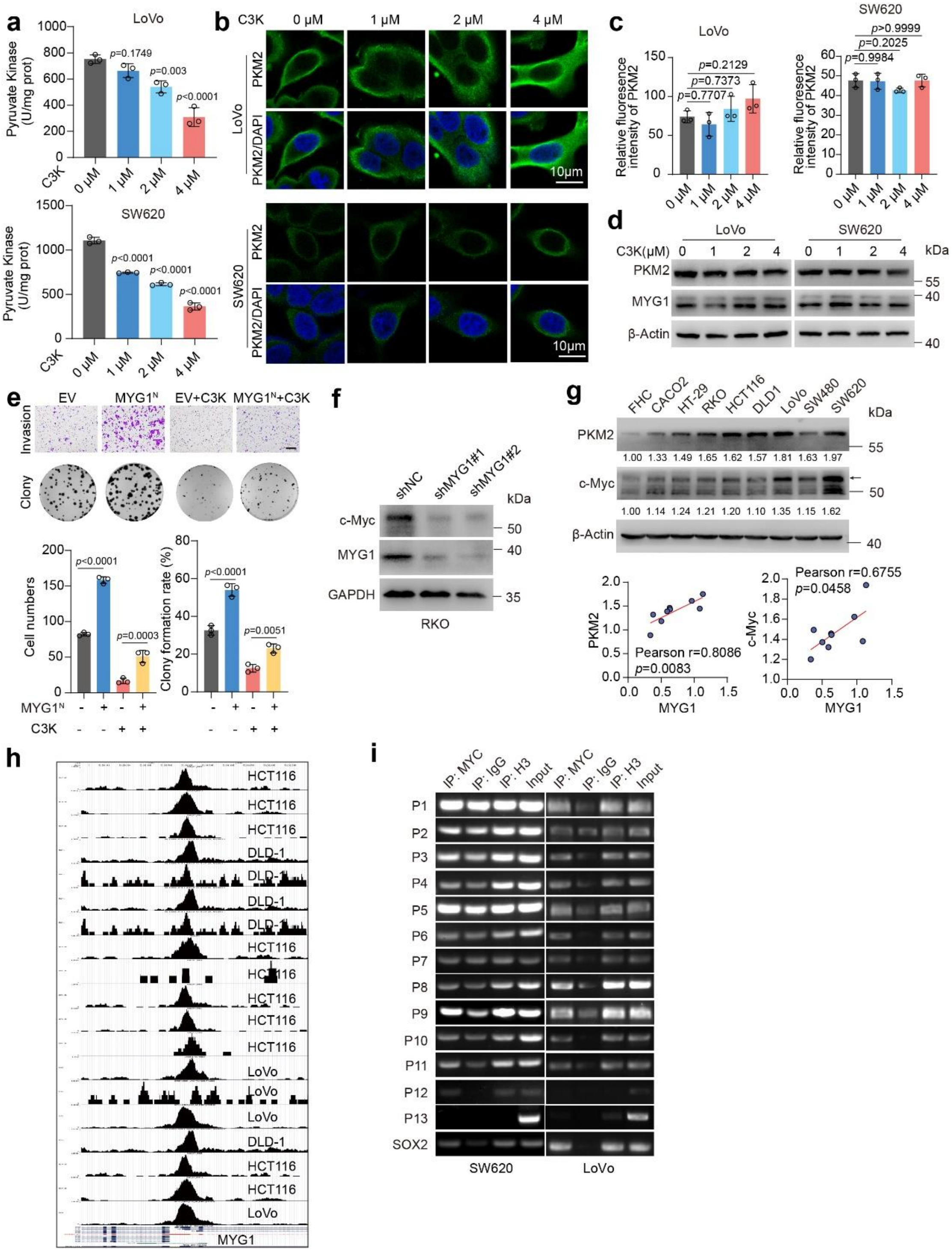

Supplementary Fig. 8. Nuclear MYG1 accelerates glycolysis through the PKM2/c-Myc signaling pathway. Related to Fig. 6. a-d Pyruvate kinase activity of PKM2 was detected in LoVo and SW620 cells treated with different concentration gradients of C3K (**a**). Expression and location of PKM2 were visualized by IF (**b**, Scale bar, 10 μm. Representative images from n = 2 independent experiments) and quantified (**c**). Expression of PKM2 and MYG1 were also detected by western blot (**d**, the samples derive from the same experiments but different gels for PKM2, and another for β-Actin and MYG1 were

processed in parrallel). **e** Representative images of transwell invasion assay (top**,** scale bar, 200 μm) and colony formation assay in HCT116 cells as indicated (top) and quantification (bottom). **f** The expression levels of c-Myc in RKO cells with MYG1 KD were detected by western blot. **g** The expression level of PKM2 and c-Myc in a normal intestinal mucosa cell line (FHC) and indicated CRC cell lines were detected by western blot (top). The correlation between the expression of MYG1 and PKM2, as well as c-Myc, was analyzed (bottom). **h** UCSC Genome Browser view of ChIP-seq profiles of c-Myc at the MYG1 gene locus in CRC cell lines from Cistrome Data Browser datasets. **i** Agarose gel blot of CHIP-qPCR assay in SW620 and LoVo cells. SOX2 was set as a positive control of ChIP. H3 antibody was set as a positive control of immunoprecipitation. One-way ANOVA, Dunnett's multiple comparisons test (**a** and **c**). Two-way ANOVA, Tukey's multiple comparisons test (**e**). Two-sided Pearson correlation (**g**). *p* value was provided in the figure. *n* = 3 independent experiments (**a, c-g** and **i**). Error bars, mean ± SD. Source data are provided as a Source Data file.

Supplementary Table 1. Correlation analysis between clinicopathological characteristics and MYG1 expression in CRC patients.

Characteristics		MYG1 expression		
	***n*=149**	**Low (%)**	**High (%)**	***p* value**
**Gender**				0.0791
Male	88	40(45.5)	48(54.5)	
Female	61	19(31.1)	42(68.9)	
**Age (years)**				0.4406
<54	74	27(36.5)	47(63.5)	
≥54	75	32(42.7)	43(57.3)	
**Tumor site**				0.1786
Proximal colon	34	9(26.5)	25(73.5)	
Distal colon	28	11(39.3)	17(60.7)	
Rectum	87	39(44.8)	48(55.2)	
**Tumor size (cm)**				0.1161
<5	80	27(33.8)	53(66.3)	
≥5	69	32(46.4)	37(53.6)	
**Tumor differentiation**				0.4148
Well	56	26(46.4)	30(53.6)	
Moderate	71	25(35.2)	46(64.8)	
Poor	22	8(36.4)	14(63.6)	
**T stage**				**0.0054**
T1-T2	39	22(56.4)	17(43.6)	
T3-T4	110	37(33.6)	73(66.4)	
**Lymph node state**				**0.0004**
Negative	82	43(52.4)	39(47.6)	
Positive	67	16(23.9)	51(76.1)	
**Distant metastasis**				**0.0194**
Negative	123	54(43.9)	69(56.1)	
Positive	26	5(19.2)	21(80.8)	

**Lymphovascular invasion**				0.2594
Negative	98	42(42.9)	56(57.1)	
Positive	51	17(33.3)	34(66.7)	
**Clinical stage**				**0.0013**
I	34	20(58.8)	14(41.2)	
II	43	22(51.2)	21(48.8)	
III	46	12(26.1)	34(73.9)	
IV	26	5(19.2)	21(80.8)	

Chi-Squared test. Statistically significant *p* values are shown in bold.

Supplementary Table 2. The primers and sequences used in the study.

Target	Type	Sequence (5'-3')
Negative Control	Si#NC	TTCTCCGAACGTGTCACGT
MYG1	qPCR primers	F: GAATCGGGACGCACAATGG
MYG1	qPCR primers	R: GGGTCCGCACAATCTCTGC
MYG1	ShRNA#1	GAGGAGTTTCTGCAGAGATTA
MYG1	ShRNA#2	GAGTTGCTCGACTTAATCCTA
MYC	SiRNA#1	GUGCAGCCGUAUUUCUACUTT
MYC	SiRNA#2	GAACACACAACGUCUUGGATT
PKM2	SiRNA#1	GGATGTTGATATGGTGTTT
PKM2	SiRNA#2	CTACCACTTGCAATTATTTGA
HSP90	SiRNA#1	GGAAAGAGCTGCATATTAA
HSP90	SiRNA#2	GGAGAAGGAACGTGATAAA
GAPDH	qPCR primers	F:TGCACCACCAACTGCTTAGC
GAPDH	qPCR primers	R: GGCATGGACTGTGGTCATGAG
ACTB	qPCR primers	F: CAGCCTTCCTTCCTGGGCATG
ACTB	qPCR primers	R: ATTGTGCTGGGTGCCAGGGCAG
ENO1	qPCR primers	F: CGGCTTTACGTTCACCTCGG
ENO1	qPCR primers	R: TCAACAGCCTTTGAGACACCCT
ALDOA	qPCR primers	F: GAGGCGTCCATCAACCTCAA
ALDOA	qPCR primers	R: GCAGCCTTCAGGTTCTCCTT
GPI	qPCR primers	F: TACTCTTCAGGAGGTCCCCG
GPI	qPCR primers	R: ACTGCAGAAGGATCCTTGGC
PFKM	qPCR primers	F: CCGTGGTTCTCGTCTCAACA
PFKM	qPCR primers	R: CGGGTGTCATATCCCAGACG
TPI1	qPCR primers	F: GGACTCGGAGTAATCGCCTG
TPI1	qPCR primers	R: TGTTGGGGTGTTGCAGTCTT
PKM2	qPCR primers	F: GGAAGTGGGCAGCAAGATCT

PKM2	qPCR primers	R: GCTCGACCCCAAACTTCAGA
GLUT1	qPCR primers	F: TGAGCATCGTGGCCATCTTT
GLUT1	qPCR primers	R: AGGCATGGAACCATTCAGGG
LDHA	qPCR primers	F: ACGTGCATTCCCGATTCCTT
LDHA	qPCR primers	R: TAGCCCAGGATGTGTAGCCT
CHIP	CHIP#SOX2	F: GCGCTGATTGGTCGCTAGAA
	CHIP#SOX2	R: CTCTGCCTTGACAACTCCTGA
	CHIP#P1	F: CCTGGGAAGCTGCATGATGT
	CHIP#P1	R: CCTGTTTAGAGGGGCATCCA
	CHIP#P2	F: GGGAAGGGAAATTCAGGGGA
	CHIP#P2	R: AAGTCCTTGGCATCCTCAGC
	CHIP#P3	F: GCCAAGGACTTCTTCAAGAGGA
	CHIP#P3	R: GAGGTGCCCCAGGAAAAACT
	CHIP#P4	F: TGGATTGCAGTGTGAACCAC
	CHIP#P4	R: GCTCCACCAAAGCACAAACA
	CHIP#P5	F: TAACCCCGACTGCACATGAG
	CHIP#P5	R: ACTTCCCCTCCTTTGACTTCC
	CHIP#P6	F: TCATTCTCACCACACCCAGC
	CHIP#P6	R: GCGCCTGGCATTGTTAATAA
	CHIP#P7	F: AAAGTTAGCCCGGGCATGGT
	CHIP#P7	R: CTCACAGCAACCTCCGTCTC
	CHIP#P8	F: TAGTCGGTCGCCTGTCCTTA
	CHIP#P8	R: GGCCTTAGCAGTAGCCTGAG
	CHIP#P9	F: CTGGTGATGGGAAGGGTCTT
	CHIP#P9	R: CCATCTCATCACCAGTCCCT
	CHIP#P10	F: GTGTGGTCGAGAGTGTGGG
	CHIP#P10	R: CAACCAGCTCTGCAGAGTGA
	CHIP#P11	F: GAGCTGGTTGCCAAGGGAA
	CHIP#P11	R: GTCCCATAAGCAGCTCCCTG
	CHIP#P12	F: TGGGACACCAATTCCTGCG
	CHIP#P12	R: GTTTGCTGCGGGATCGTTTT
	CHIP#P13	F: GAATCGGGACGCACAATGG
	CHIP#P13	R: GGGTCCGCACAATCTCTGC
